# Chromosome-level genome assembly of the diploid oat species *Avena longiglumis*

**DOI:** 10.1038/s41597-024-03248-6

**Published:** 2024-04-22

**Authors:** Qing Liu, Gui Xiong, Ziwei Wang, Yongxing Wu, Tieyao Tu, Trude Schwarzacher, John Seymour Heslop-Harrison

**Affiliations:** 1 State Key Laboratory of Plant Diversity and Specialty Crops / Guangdong Provincial Key Laboratory of Applied Botany, South China Botanical Garden, Chinese Academy of Sciences, Guangzhou, 510650 China; 2 South China National Botanical Garden, Guangzhou, China; 3 University of Chinese Academy of Sciences, Beijing, China; 4 School of Biology and Agriculture, Shaoguan University, Shaoguan, China; 5 College of Agriculture, South China Agricultural University, Guangzhou, China; 6 University of Leicester, Department of Genetics and Genome Biology, Institute for Environmental Futures, Leicester, UK

**Keywords:** Plant evolution, Phylogenetics, Genome evolution

## Abstract

Diploid wild oat *Avena longiglumis* has nutritional and adaptive traits which are valuable for common oat (*A*. *sativa*) breeding. The combination of Illumina, Nanopore and Hi-C data allowed us to assemble a high-quality chromosome-level genome of *A*. *longiglumis* (ALO), evidenced by contig N50 of 12.68 Mb with 99% BUSCO completeness for the assembly size of 3,960.97 Mb. A total of 40,845 protein-coding genes were annotated. The assembled genome was composed of 87.04% repetitive DNA sequences. Dotplots of the genome assembly (PI657387) with two published ALO genomes were compared to indicate the conservation of gene order and equal expansion of all syntenic blocks among three genome assemblies. Two recent whole-genome duplication events were characterized in genomes of diploid *Avena* species. These findings provide new knowledge for the genomic features of *A. longiglumis*, give information about the species diversity, and will accelerate the functional genomics and breeding studies in oat and related cereal crops.

## Background & Summary

Common oat (*Avena sativa* L.) and its wild relatives (2*x*, 4*x*,and 6*x*) are members of the Aveneae tribe (Poaceae). Clinical studies have shown the beneficial effects of consuming oats that can reduce serum cholesterol and cardiovascular disease, attributed to the soluble β-glucan component^1^. Oats also exhibit a favourable glycaemic index, with a low value and slow carbohydrate breakdown. Plant oils derived from cereal seeds are vital agricultural commodities used for food, feed, and fuel. Oat endosperm has between 6–18% oil content, which is significantly higher than other cereals [averaging 2.41% in barley (*Hordeum vulgare*) and 2.18% in wheat (*Triticum aestivum*)]^2,3^. The high oil content of oat grain suggests a possible important use for food oils and in animal feeds^4^. Despite the unique composition, global oat production has steadily declined over the past 50 years to 25 million tons in 2023 (http://www.fao.org/faostat/), suggesting the genetic improvement has lagged behind major cereal crops such as rice, wheat, and maize, making the crop less desirable to grow. There are therefore likely to be substantial opportunities for improvement of oat varieties.

**Fig. 1:**
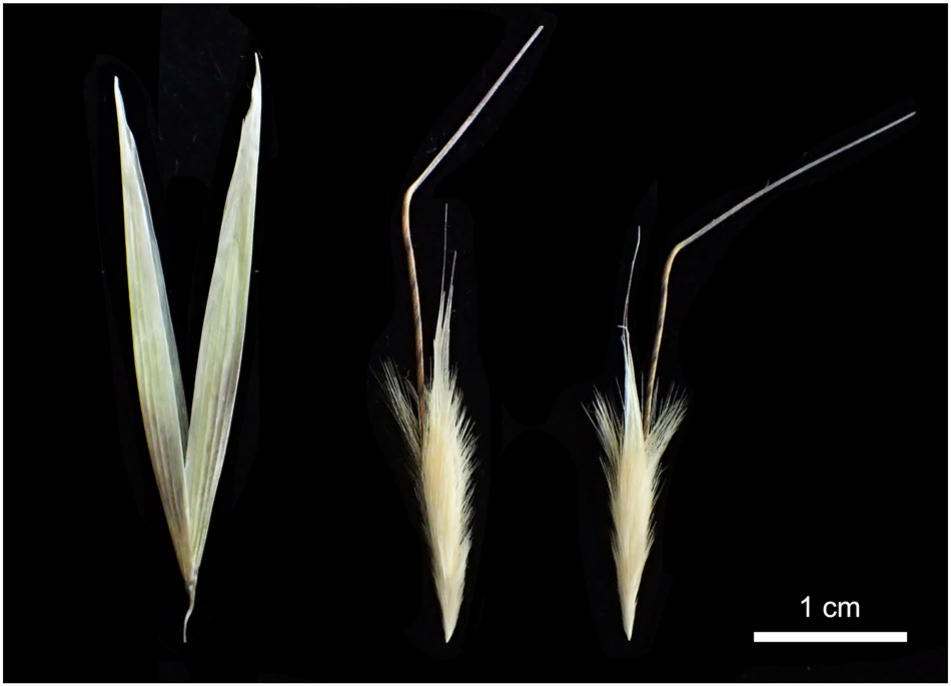
The spikelet of *Avena longiglumis*. Two glumes nearly equal in length (left), the first (middle) and the second (right) florets disarticulated with 2–3 mm awl-shaped callus at the floret base together with 8–12 mm bristles at the lemma tip. Scale bar, 1 cm.

Not least due to the large genome size of *A*. *sativa* (10.3 Gb)^5^, oat genomic research lags behind that of other crops such as rice (*Oryza sativa*)^6^, sorghum (*Sorghum bicolor*)^7^ or foxtail millet (*Setaria italica*)^8^. There is an urgent need for the characterization, exploitation and utilization of wild oat germplasm resources for oat and related crop breeding^9,10^. A diploid genome of *A*. *longiglumis* Durieu (Fig. 1) reveals novelty in target genes and regulatory sequences, such as those for β-glucan synthesis, high linoleic content in grains, drought-adapted phenotypes, and resistance to crown rust disease^11^. The rapidly developing field of structural variation requires multiple high-quality chromosome-scale assemblies to show the nature of intraspecific variation (individual, variety or populations), polymorphisms within and between diploid species and their related species, and generation of recent structural variations in polyploid species derived from diploid ancestors.

**Fig. 2:**
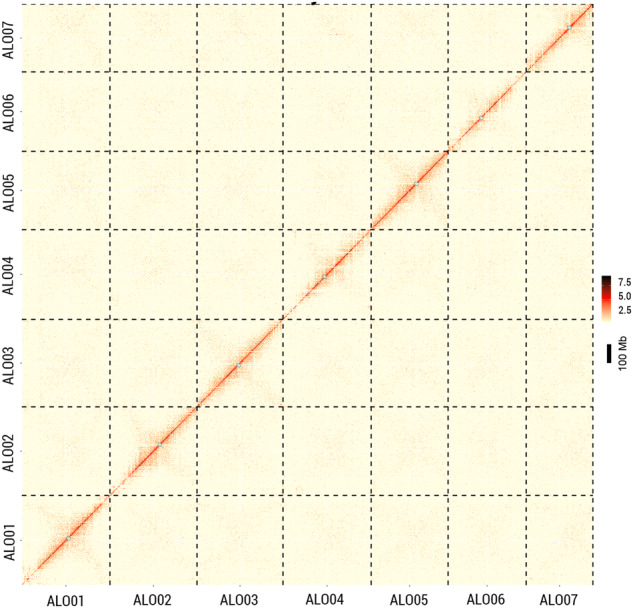
Genome-wide chromatin interaction heatmap (100 kb bins) of diploid *A. longiglumis* (ALO, PI657387) based on Hi-C data showing chromosome-scale continuity of the assembly. Small shaded circles denoted the centromeric locations.

**Fig. 3:**
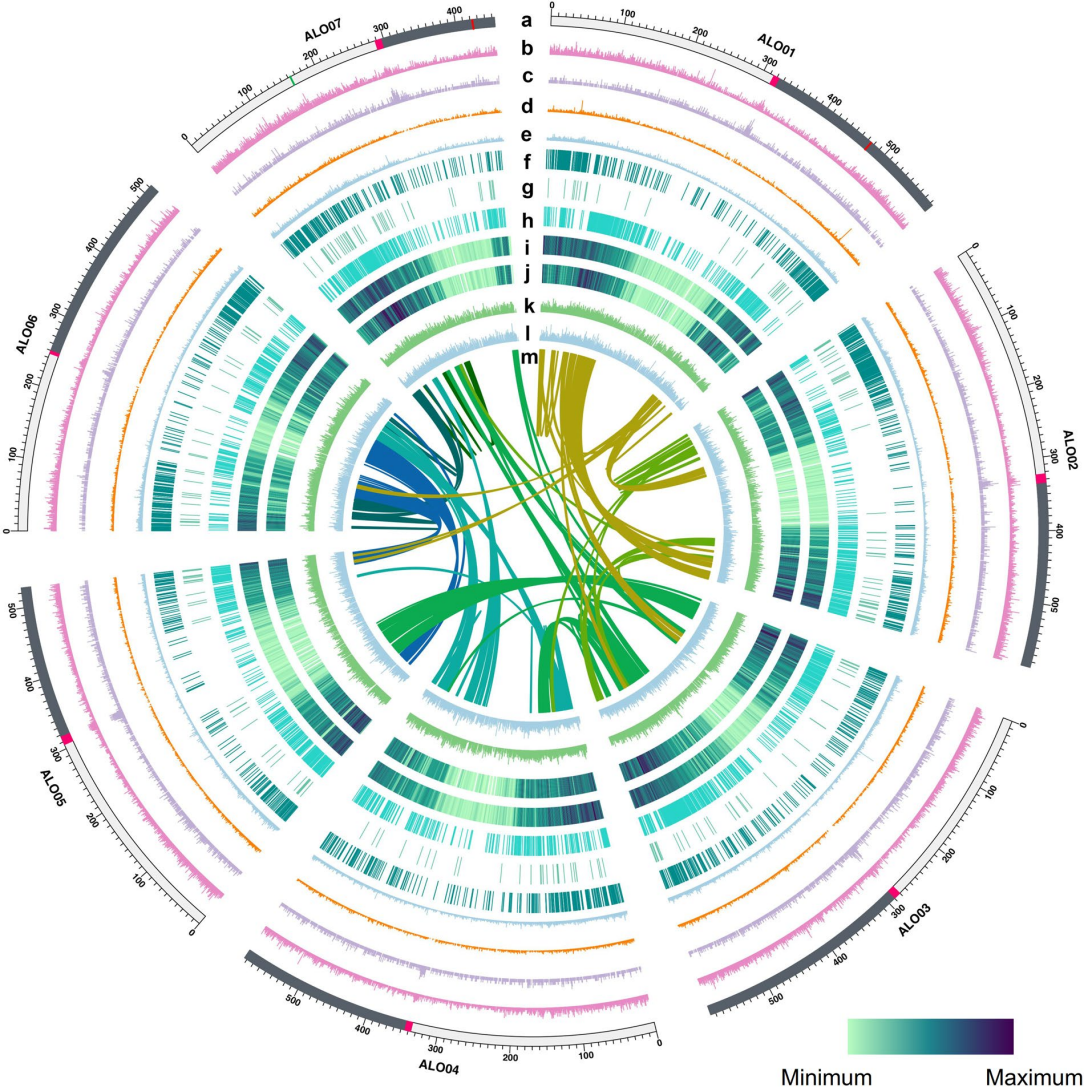
Genomic features of *Avena longiglumis* PI657387. (**a**) Seven chromosomes (scale in 100 Mb) with pink, green and red regions denoting centromere, 5 S (ALO07) and 45 S (ALO01 and ALO07) rDNA positions. (**b**) Transposable element (TE) density. (**c**) Long-terminal repeat TE density. (**d**) Long interspersed nuclear element (LINE) density. (**e**) *Helitron* density (cyan). (**f**) Expanded gene locations. (**g**) Contracted gene locations. (**h**) Single copy orthologue gene locations. (**i**) High-confidence gene locations. (**j**) Purified selection gene (P-value ≤ 0.05) locations. (**k**) Gene expression profiling in ALO roots. (**l**) Gene expression profiling in ALO leaves. (**m**) Inter-chromosomal synteny. **b, d**–**h** & **k**–**l**: 100 bp bins; **c**: 1 Mb bins; **i**–**j**: 3 kb bins.

This study utilized a combination of Illumina, Oxford Nanopore Technology (ONT) sequencing, and chromosome conformation capture (Hi-C) data to create a superior chromosome-scale genome assembly of diploid *A*. *longiglumis* (ALO; Fig. S1). Its genome assembly had a length of approximately 3,960.97 Mb (Table 1 and S1), which is slightly smaller than the genome size estimated by k-mer analysis (Fig. S2). Through scaffolding contigs into seven super-scaffolds, the 98.84% of reads were anchored. As observed in the Hi-C heatmap, the seven super-scaffolds were mapped to the corresponding seven pseudo-chromosomes (Fig. 2). Among *A*. *longiglumis* genome sequences, 87.04% were classified as known repetitive DNA elements (Table 2), showing increased density in broad centromeric regions (Fig. 3 circle b). Compared to the published assembly results of tetraploid *A*. *insularis* and hexaploid oat genomes^5,9^, the diploid *A. longiglumis* genome in this study exhibits superior sequence continuity, as evidenced by higher contig N50 value of 12.68 Mb and scaffold N50 value of 527.34 Mb, respectively (Table 3), indicating a high assembly quality of the diploid genome, ensuring the reliability of subsequent research.

**Table 1:** Genome assembly statistics and gene predictions in the *Avena longiglumis* genome.

Features	Number	Size
*Assembly features*		
Predicted genome size based on k-mer		3,965,670,000 bp
Assembly size		3,960,965,270 bp
Total length of seven pseudo-chromosomes		3,847,578,604 bp
Scaffold N50 length		527,343,613 bp
Scaffold N90 length		6,968,329 bp
Number of scaffolds (>N90)		9
Longest scaffold (bp)		594,546,470 bp
Contig N50 length		12,682,464 bp
Longest contig		99,445,397 bp
*Repetitive DNAs*		
Retrotransposons		3,198,067,781 bp (80.74%)
DNA transposons		137,389,012 bp (3.47%)
Total repeats		3,447,484,807 bp (87.04%)
*Gene annotation*		
High-confidence (HC) genes	33,271	115,042,134 bp
Low-confidence (LC) genes	7,574	18,590,004 bp
Total genes	40,845	133,632,138 bp
Average length of each gene		3,272 bp
Non-coding RNAs	16,439	2,222,342 bp

**Table 2:** Repetitive DNA composition of the *Avena longiglumis* genome.

Repeat type		Super family	Family	Repeat sequences (bp)	Copy number	Repeat fraction	Genome fraction
Transposable elements							
Class I (Retrotransposons)							
LTR	Gypsy			2,045,839,268	1,127,011	59.34%	51.65%
	Copia			1,035,647,971	575,382	30.04%	26.15%
	Unknown LTR			77,372,748	61,272	2.24%	1.95%
	Other LTR			213,586	628	0.01%	0.01%
Total LTR- Retrotransposons				3,159,073,573	1,764,293	91.63%	79.76%
Non-LTR	LINE	L1		38,994,208	42,780	1.13%	0.98%
Total Class I retrotransposons				3,198,067,781	1,807,073	92.76%	80.74%
Class II (DNA transposons)-Subclass 1							
	Tc1_Mariner			21,858,891	69,956	0.63%	0.55%
	CACTA			29,566,991	75,699	0.86%	0.75%
	Mutator			25,388,681	82,292	0.74%	0.64%
	PIF_Harbinger			9,194,639	31,402	0.27%	0.23%
	hAT			7,600,354	22,266	0.22%	0.19%
Class II (DNA transposons)-Subclass II							
	Helitron			43,779,456	117,248	1.27%	1.11%
Total Class II DNA transposons				137,389,012	398,863	3.99%	3.47%
Total transposable elements				3,335,456,793	2,205,936	96.75%	84.21%
Tandem and simple sequence repeats				11,144,119	162,888	0.32%	0.28%
Other repeats				100,883,895	369,975	2.93%	2.55%
Total repetitive DNAs				3,447,484,807	2,728,799	100%	87.04%

**Table 3:** Summary of genome assemblies of *Avena longiglumis* of this study and published tetraploid *A. insularis* and hexaploid *A. sativa*. –: unavailable data.

Species	*Avena longiglumis* PI 657387	*A.insularis* BYU209^5^	*A.sativa* cv. Sang	*A. insularis* CN108634	*A. sativa* ssp. *nuda cv. Sanfensan*^9^	*A. sativa_*OT3098v.2
Number of contigs	2,381	6,523	1,823,168	2,732	436	1,343
Number of scaffolds	414	15	22	—	—	84
Assembled sequences	3,960,965,270 bp	7,256,293,586 bp	11,012,379,496 bp	7,519,018,440 bp	10,757,433,345 bp	10,839,200,031 bp
Contig N50 length	12.682 Mb	5.157 Mb	21.001 kb	5.637 Mb	75.273 Mb	71.000 Mb
Scaffold N50 length	583.925 Mb	481.348 Mb	490.397 Mb	—	—	374.00 Mb
BUSCO	99.00%	99.60%	99.40%	99.32%	99.44%	99.38%

**Fig. 4:**
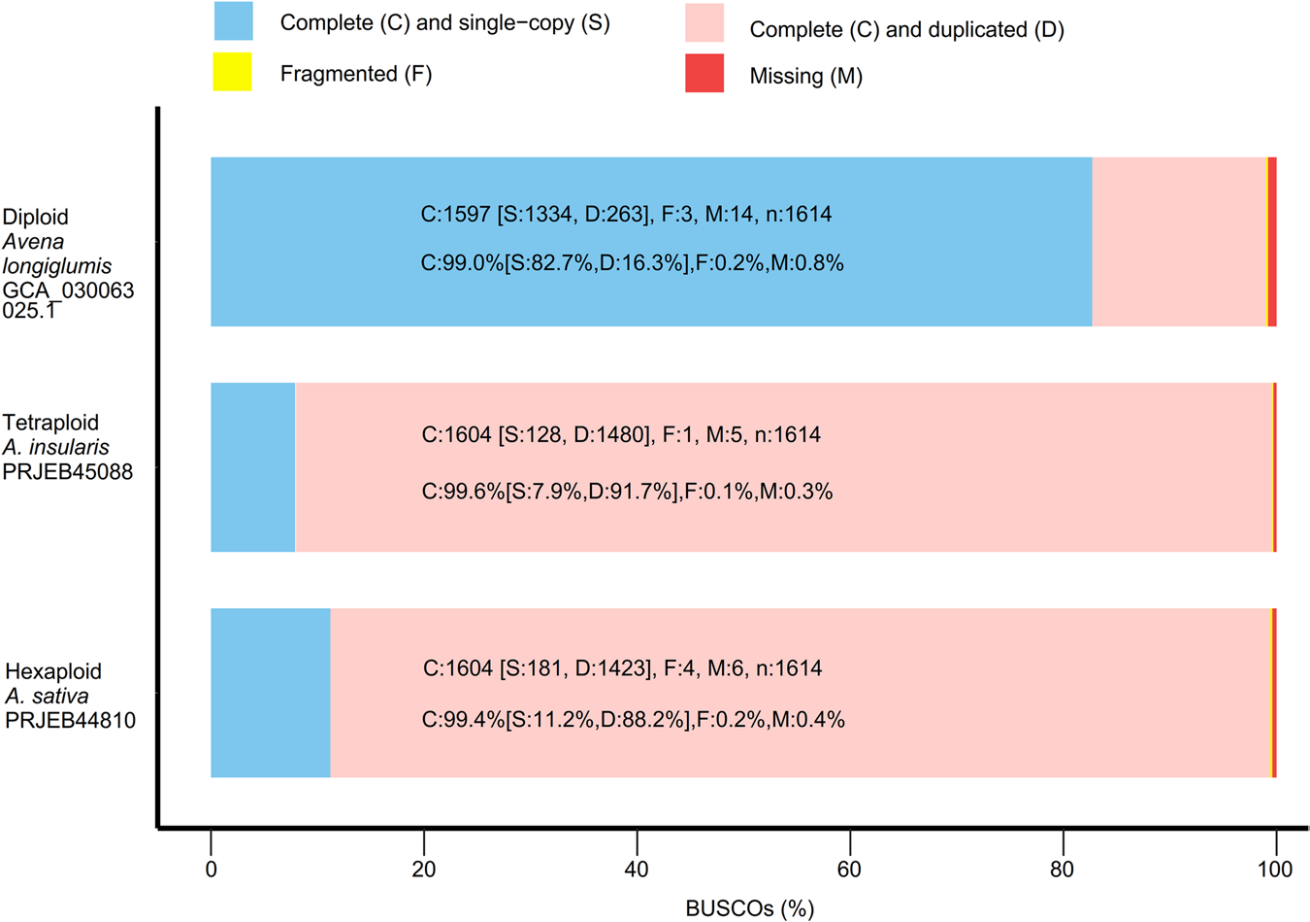
BUSCO scores of the assembled genomes of *Avena longiglumis*, *A. insularis* (Kamal *et al*.^22^), and *A. sativa* (Kamal *et al*.^22^). Our *A. longiglumis* genome assembly stored on GenBank https://identifiers.org/ncbi/insdc.gca: GCA_030063025.1 (2023); Genome assemblies of *A. insularis* and *A. sativa* from the European Nucleotide Archive (ENA) under accession numbers PRJEB45088 and PRJEB44810, respectively.

The BUSCO^12^ results revealed the retrieval of 99.0% of the complete single-copy genes, of which 16.3% were duplicated, indicating high genome assembly completeness of our *A. longiglumis*_CN58138 (Table S2). Compared to other diploid assemblies of *A. longiglumis*_CN58138 (93.0%) and *A. eriantha* (94.0%) (Extended Data Fig. 2a of ref. ^5^), our diploid *A. longiglumis*_PI657387 genome exhibited a higher proportion of complete orthologous genes, comprising 99.0% of the genome assembly (Fig. 4). Compared to tetraploid *A. insularis* (7.9%) and hexaploid *A. sativa* (11.2%), the *A. longiglumis* genome in our study exhibits a higher proportion of single-copy orthologous genes, comprising 82.7% of the genome assembly (Fig. 4). In addition, the fragmented genes in this diploid genome display a similarity (0.2%) to those found in *A*. *sativa*.

**Fig. 5:**
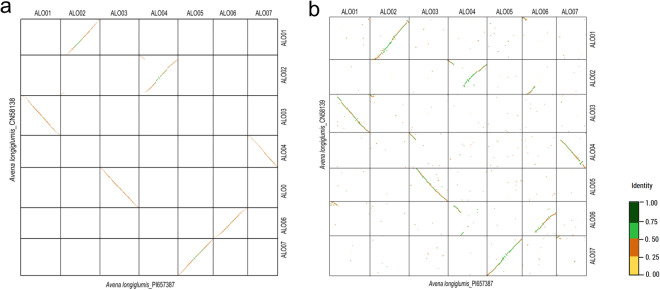
Pairwise comparisons of dotplots for three *Avena longiglumis* (ALO) genome assemblies and the diploid *Avena* species genomes. (**a**) ALO_PI657387and ALO_CN58138 (Kamal *et al*.^22^). (**b**) ALO_PI657387 and ALO_ CN58139 (Peng *et al*.^9^). The dotplots provide insights into the conservation of gene order and the genomic rearrangements among three *A. longiglumis* genome assemblies. The x- and y-axes represent the genomic coordinates of each species.

A total of 40,845 protein-coding genes were annotated for *A. longiglumis* using databases of NCBI NR (Non-redundant protein)^13^, EggNOG (Evolutionary genealogy of genes: non-supervised orthologous groups)^14^, Pfam (Pfam protein families)^15^, COG (Clusters of orthologous groups)^16^, SwissProt (Swiss Institute of Bioinformatics and Protein Information Resource)^17^, GO (Gene ontology)^18^, KOG (EuKaryotic orthologous groups)^19^, KEGG (Kyoto encyclopedia of genes and genomes)^20^, PlantTFDB (Plant transcription factor)^21^, and CAZy (Carbohydrate-Active enZYmes)^22^ (Table S3). Dotplots of our *A. longiglumis* assembly were compared with two published genomes of *A. longiglumis*^5,9^, indicating the conservation of gene order and equal expansion of all syntenic blocks among three ALO genome assemblies (Fig. 5a,b).

## Methods

### Plant Materials

Young leaf samples were collected from an *A. longiglumis* plant (ALO, accession PI 657387; US Department of Agriculture at Beltsville, https://www.ars-grin.gov/, originally collected in Morocco) grown in climatic box conditions (16 h light / 8 h dark and day/night temperatures of 25°C/15°C) at the South China National Botanical Garden, Guangzhou, China. Young leaves were collected for DNA isolation and whole-genome sequencing. The leaves and roots were collected for RNA-sequencing (RNA-seq) and transcriptome assembly. The samples were immediately flash-frozen in liquid nitrogen after harvest, and stored at −80 °C for subsequent nucleic acid extraction. The extraction and purification of RNA were carried out utilizing the Qiagen RNeasy Plant Mini Kit (Qiagen, CA, USA), following the instructions of the manufacturer, one of 8 Gb and one of 10 Gb pair-end read data were obtained. A total of 511.4 Gb Oxford Nanopore Technology (ONT) long reads (~128.9 × coverage), 435.6 Gb Hi-C reads (~109.8 × coverage), 268.6 Gb (~67.7 × coverage) paired-end Illumina reads, and 99.0 Gb RNA-seq reads were generated for the genome assembly, genome survey, and transcriptome assembly (Table S1).

### Illumina sequencing and genome survey

Pair-end genome sequencing with a 350 bp insert size used Illumina TruSeq^®^ Nano DNA library preparation kit (Illumina, San Diego, CA, USA) and libraries were sequenced on an Illumina NovaSeq 6000 platform (Table S1). Fastp v.0.23.2^23^ was utilized to remove contaminants, Illumina adapters, and low-quality reads. The 268.60 Gb clean data were processed via Kmerfreq_AR v.2.0.4^24^. The 17-bp k-mers with Illumina reads counted using Jellyfish v.2.2.6^25^ with default parameters. The genome size of 3.966 Gb, a heterozygosity of 0.48%, and repeat content were estimated using GenomeScope v.2.0^26^ (Fig. S2).

### ONT sequencing and genome assembly

The genomic DNA (10 μg) was broken into fragments around 10–50 kb long with the use of a g-TUBE device (Covaris, Inc., MA, USA) and size selection with BluePippin (Sage Science, Inc., MA, USA). To prepare the ONT PromethION (Genome Centre of Grandomics, Wuhan, China) sequencing libraries, DNA end repair was carried out by utilizing the NEBNext End Repair/dA-Tailing Module (New England Biolabs, MA, UK), and the ligation sequencing kit (SQK-LSK109, ONT, UK) (Table S1).

ONT reads were subjected to self-correction using three tools, NextDenovo v.2.4.0 (https://github.com/Nextomics/NextDenovo), wtdbg2.huge v.1.2.8^27^ and SMARTdenovo v.1.0.0^28^. The corrected reads were then passed on to NextDenovo for additional read correction. Subsequently, we evaluated several parameters and detected that utilizing the corrected reads in combination with SMARTdenovo v.1.0.0^28^ and assembler parameters “-c 3” and “-k 11” produced desired outcomes by generating a preliminary assembly. The contigs were polished with ONT raw data thrice using NextPolish v.1.01^29^ and four times with filtered Illumina reads.

### Hi-C sequencing and chromosome-level genome assembly

For Hi-C sequencing, 3-week-old leaves of *A. longiglumis* seedlings were fixed in 2% formaldehyde solution to obtain nuclear/chromatin samples. *Dpn*II enzyme (Cat. E0543L, NEB, UK) was utilized to digest these fixed tissues. Hi-C libraries were then constructed and sequenced on the Illumina Novaseq 6000 platform to generate 150 bp paired-end reads (Table S1). High-quality reads were extracted and aligned to the reference genome assembly using Bowtie2 v.2.3.2^30^. Juicer v.2.0^31^ was utilized to create a de-duplicated listing of alignments of Hi-C reads to the draft *A*. *longiglumis* assembly. HiC-Pro v.2.7.8^32^ was used to determine the ligation site for each unmapped read, after which the 5’ fragments were aligned to the genome assembly.

A single alignment file was generated by merging the results of both mapping steps, and low-quality reads were discarded, which included reads with multiple matches, singletons, and mitochondrial DNA. Valid pairs of interaction were employed in scaffolding the assembled contigs into 7 pseudo-chromosomes utilizing the LACHESIS pipeline^33^. The quality and completeness of the genome assembly were evaluated by utilizing BUSCO v.5.4.6^12^ (Table S2). In addition, the chromosome matrix was depicted as a heatmap that manifested diagonal patches of robust linkage.

### Identification and characterization of repetitive elements

*De novo* repeat prediction of the ALO assembly was carried out by EDTA v.1.7.0 (Extensive *de-novo* TE Annotator)^34^, which was composed of eight software. LTRharvest^33,34^, LTR_FINDER_parallel v.1.2^35^, LTR_retriever v. 2.9.0^36^ (it was incorporated to identify LTR retrotransposons); Generic Repeat Finder v.1.7.0^37^ and TIR-Learner v.1.7.0^38^ were included to identify TIR transposons; HelitronScanner v.1.0^39^ was identified Helitron transposons; RepeatModeler v.2.0.2a^40^ was used to identify transposable elements (TEs, such as LINEs); Finally, RepeatMasker v.4.1.1^41^ was used to annotate fragmented TEs based on homology to structurally annotated TEs. In addition, TEsorter v.1.1.4^42^ was used to identify TE-related genes.

### Gene prediction and functional annotation

Gene structure prediction relied on three distinct approaches that were applied, including *ab initio* prediction, homology-based prediction, and RNA-seq-assisted prediction^43^. The *de novo*-based gene prediction was carried out using Augustus v.3.4.0^44^ with default parameters, to predict *A. longiglumis*-assembled genes. Furthermore, the homology-based prediction was performed by GeMoMa v.1.6.1^45^ with default parameters, utilizing filtered proteins from genomes of six species (*Arabidopsis thaliana*^46^, *Brachypodium distachyon*^47^, *Hordeum vulgare*^48^, *Sorghum bicolor*^7^, *Triticum aestivum*^49^ and *Zea mays*^50^). The RNA-seq-based gene prediction was executed using TransDecoder v.5.5.0^51^. High-confidence (HC) genes refer to both homology-based prediction supported by ≥ two species (1,083) and by RNA-seq-assisted prediction if the FPKM (Fragments Per Kilobase of exon model per Million mapped fragments) value > 0 (32,188). The predicted gene structures from each of these three approaches were integrated into consensus gene models using EVidenceModeler v.1.1.1^52^. The resulting gene models were then filtered to obtain a precise gene set, whereby genes with transposable element sequences were removed using TransposonPSI v.1.0.0 (http://transposonpsi.sourceforge.net/).

Functional annotation was performed for the predicted protein-coding genes via comparing with public databases including NCBI NR^13^, EggNOG^14^, Pfam^15^, COG^16^, SwissProt^17^, GO^18^, KOG^19^, KEGG^20^, PlantTFDB^21^, and CAZy^22^ (Table S3). Protein sequences were aligned to NCBI NR^13^, SwissProt^17^ and KOG^19^ by BLASTP v.2.10.1^53^ (E-value ≤ 1e-15). EggNOG^14^, Pfam^15^, and COG^16^ annotations were performed with eggNOG v.5.0^14^. GO^18^ ID for each gene were determined using Blast2GO v.1.44^54^. Genes were mapped to KEGG database^20^ (Fig. S3). Additionally, transcription factor annotation was carried out using PlantTFDB v.5.0^21^, while gene annotation used CAZy^22^ (Table S3).

### Non-coding RNA annotation

The prediction of the non-coding RNA gene set (ncRNA) was carried out across the genome. Initially, the data was aligned with the noncoding database of Rfam library v.11.0^55^, for the annotation of genes encoding various non-coding RNAs including small nuclei RNAs (snRNAs), ribosomal RNAs (rRNAs), and microRNAs (miRNAs). The transfer RNA (tRNA) sequences were subsequently identified using tRNAscan-SE v.2.0^56^ (Table 1).

### Pairwise comparisons of genome assemblies

To create the dotplots of *A. longiglumis*, the reference sequence of CN58138^5^ and CN58139^9^ were aligned with the *de novo* assembly of PI 657387 using Minigraph v. 2.25^57^, respectively, with the ‘-ax asm5’ option, resulting in a PAF alignment file. The PAF file was uploaded to D-Genies v.1.5.0^58^ to create the dotplot using their default setting. Dotplots of the assembly (accession PI657387) were compared with two published genomes of *A. longiglumis*, indicating the conservation of gene order and equal expansion of all syntenic blocks among three genome assemblies (Fig. 5a,b).

## Data Records

Sequencing reads for *Avena longiglumis* are available on the NCBI Sequence Read Archive (SRA) https://identifiers.org/ncbi/insdc.sra: SRR19279518^59^ for genome survey data; SRR19279519-SRR19279520 and SRR19279522-SRR19279531^59^ for ONT data; SRR19279511-SRR19279517, SRR19279521, and SRR19279532-SRR19279533^59^ for Hi-C data; and SRR24234795-SRR24234797 and SRR24234802-SRR24234804^60^ for RNA sequencing data. Genome assembly for *A*. *longiglumis* is available on the GenBank https://identifiers.org/ncbi/insdc.gca: GCA_030063025.1^61^.

## Technical Validation

The chromosome-level genome assembly was 3,960.97 Mb with a scaffold N50 of 527.34 Mb. The interaction contact pattern was organized around the principal diagonal in the Hi-C heatmap (Fig. 2), directly supporting the accuracy of the chromosome assembly. The completeness of the final assembled genome was assessed using BUSCO v.5.4.6^12^ by searching embryophyta_odb10 databases. The results revealed the retrieval of 99.0% of the complete single-copy genes, of which 16.3% were duplicated. Only 0.2% of BUSCO genes were fragmented, and 0.8% were missing from the *A. longiglumis* genome (Fig. 4).

### Supplementary information

Supplementary Information

## Data Availability

Parameters of software tools involved in the methods are described below:1) Fastp: version 0.23.2, default parameters;2) Kmerfreq_AR: version 2.0.4, parameters: (k-mer size of 17);3) Jellyfish: version 2.2.6, parameters: (count -m 17 -s 10 G -t 10 -C);4) GenomeScope: version 2.0, parameters: (k-mer size of 17, read length of 100, maximum k-mer coverage of 1000);5) NextDenovo: version 2.4.0, parameters: (read_cutoff = 3k, seed_cutoff = 27k, blocksize = 5 g);6) wtdbg 2.huge: version 1.2.8, parameters: (wtdbg-1.2.8 -k 0 -p 21 -S 2, wtdbg-cns -c 0 -k 13, kbm-1.2.8 -k 0 -p 19 -S 2 -O 0, wtdbg-cns -k 11 -c 3);7) SMARTdenovo: version 1.0.0, parameters: (-c 3 and -k 11);8) NextPolish: version 1.01, default parameters;9) Bowtie2: version 2.3.2, parameters: (-end-to-end,–very-sensitive –L 30);10) Juicer: version 2.0, default parameters;11) HiC-Pro: version 2.7.8, default parameters;12) LACHESIS: latest version, parameters: (CLUSTER MIN RE SITES = 100; CLUSTER MAX LINK DENSITY = 2.5; CLUSTER NONINFORMATIVE RATIO = 1.4; ORDER MIN N RES IN TRUNK = 60; ORDER MIN N RES IN SHREDS = 60);13) BUSCO: version 5.4.6, parameters: (embryophyta_odb10);14) EDTA: version 1.7.0, parameters: (sudo docker run -it -v $PWD:/in -w /in oushujun/edta:1.9.5);15) LTRharvest: lastest version, parameters: (-minlenltr 100 -maxlenltr 7000 -mintsd 4 -maxtsd 6 -motif TGCA -motifmis 1 -similar 85 -vic 10 -seed 20 -seqids yes);16) LTR_FINDER_parallel: version 1.2, default parameters;17) LTR_retriever: version 2.9.0, default parameters;18) Generic Repeat Finder: version 1.7.0, default parameters;19) TIR-Learner: version 1.7.0, default parameters;20) HelitronScanner: version 1. 0, default parameters;21) RepeatModeler: version 2.0.2a, default parameters;22) RepeatMasker: version 4.1.1, parameters: (-pa 30 -lib –no_is -poly -html -gff -dir masker);23) TEsorter: version 1.1.4, default parameters;24) Augustus: version 3.4.0, default parameters;25) GeMoMa: version 1.6.1, default parameters;26) TransDecoder: version 5.5.0, parameters: (-G universal, -m 100);27) EVidenceModeler: version 1.1.1, default parameters;28) TransposonPSI: version 1.0.0, default parameters;29) BLASTP: version 2.10.1, parameters: (-outfmt 6, -evalue 1e-15);30) eggNOG: version 5.0, default parameters;31) Blast2GO: version 1.44, default parameters;32) PlantTFDB: version 5.0, default parameters;33) CAFE: version 4.2.1, default parameters;34) Rfam library: version 11.0, default parameters;35) tRNAscan-SE: version 2.0, default parameters;36) Minigraph2: version 2.25 (r1173), parameters: (-ax asm5);37) D-GENIES: version 1.5.0, default parameters;
