# Supplementary material for: Chromosome-level genome assembly of the diploid oat species Avena longiglumis
Source: Sci Data. 2024 Apr 22;11:412. doi: 10.1038/s41597-024-03248-6 (PMC11035610; doi:10.1038/s41597-024-03248-6)
Supplement: Supplementary file 1 — Supplementary Information [file 41597_2024_3248_MOESM1_ESM.pdf]

Supplementary Information

Chromosome-level genome assembly of the diploid oat species *Avena longiglumis*

Qing Liu[1,2*], Gui Xiong[1,3], Ziwei Wang[4], Yongxing Wu[5], Tieyao Tu[1,2], Trude Schwarzacher[1,2,6], John Seymour Heslop-Harrison[1,2,6*]

[1] State Key Laboratory of Plant Diversity and Specialty Crops / Guangdong Provincial Key Laboratory of Applied Botany, South China Botanical Garden, Chinese Academy of Sciences, Guangzhou 510650, China.

[2] South China National Botanical Garden, Guangzhou, China.

[3] University of Chinese Academy of Sciences, Beijing, China.

[4] School of Biology and Agriculture, Shaoguan University, Shaoguan, China.

[5] College of Agriculture, South China Agricultural University, Guangzhou, China.

[6] University of Leicester, Department of Genetics and Genome Biology, Institute for Environmental Futures, Leicester, UK.

[*] Corresponding authors: Qing Liu (liuqing@scib.ac.cn); John Seymour Heslop-Harrison (phh4@le.ac.uk)

Contents

Supplementary Figure S1. Strategy for sequencing, assembly and annotation of the *Avena longiglumis* genome.

Supplementary Figure S2. The result of k-mer analysis.

Supplementary Figure S3. KEGG enrichment of expanded genes in four *Avena* species.

Supplementary Table S1. Summary of sequencing libraries of *Avena longiglumis* included in the study.

Supplementary Table S2. Evaluation of gene space completeness by BUSCO analysis in the *A. longiglumis* genome assembly.

Supplementary Table S3. Statistics of gene function annotation of the *A. longiglumis* genome.

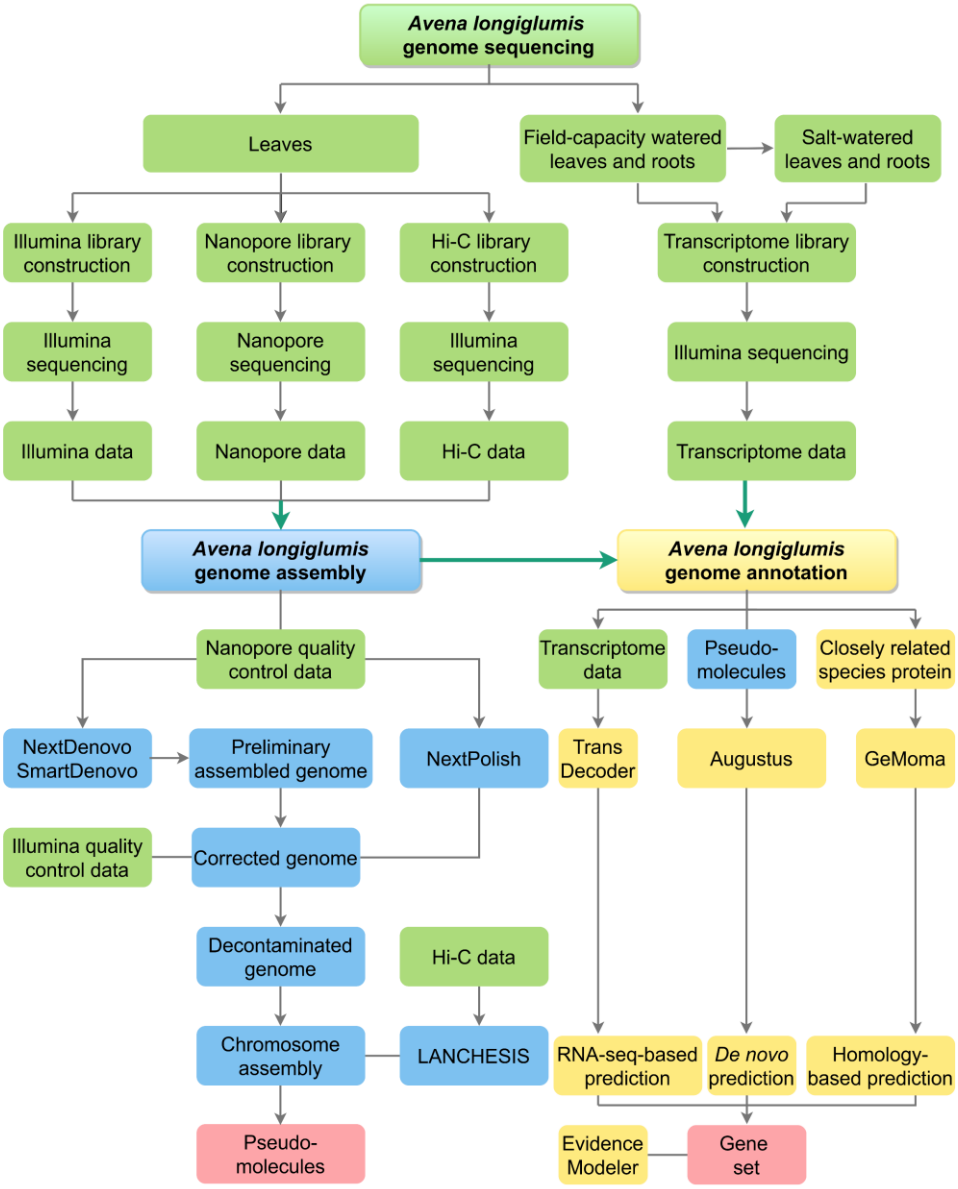

Supplementary Figure S1. Strategy for sequencing, assembly and annotation of the *Avena longiglumis* genome.

a

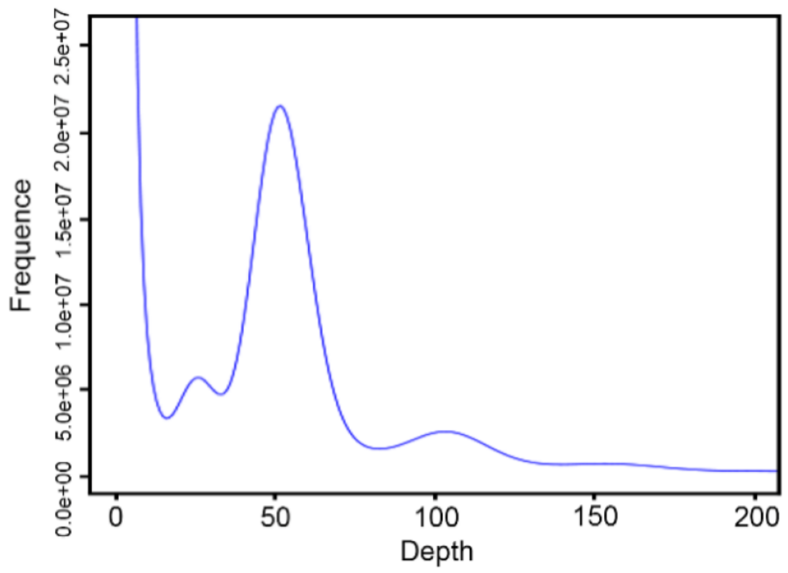

b

K-mer	Depth	Number of k-mers	Genome size (bp)	Heterogygous rate (%)	Repeat rate (%)
17	52	208,164,726,959	3,965,670,000	0.48	82.08

Supplementary Figure S2. The result of k-mer analysis. (**a**) The 17-mer frequency distribution in *A. longiglumis* genome. The x-axis is the k-mer depth, and y-axis represents the frequency of the k-mer for a given coverage. (**b**) Statistics of k-mer analysis.

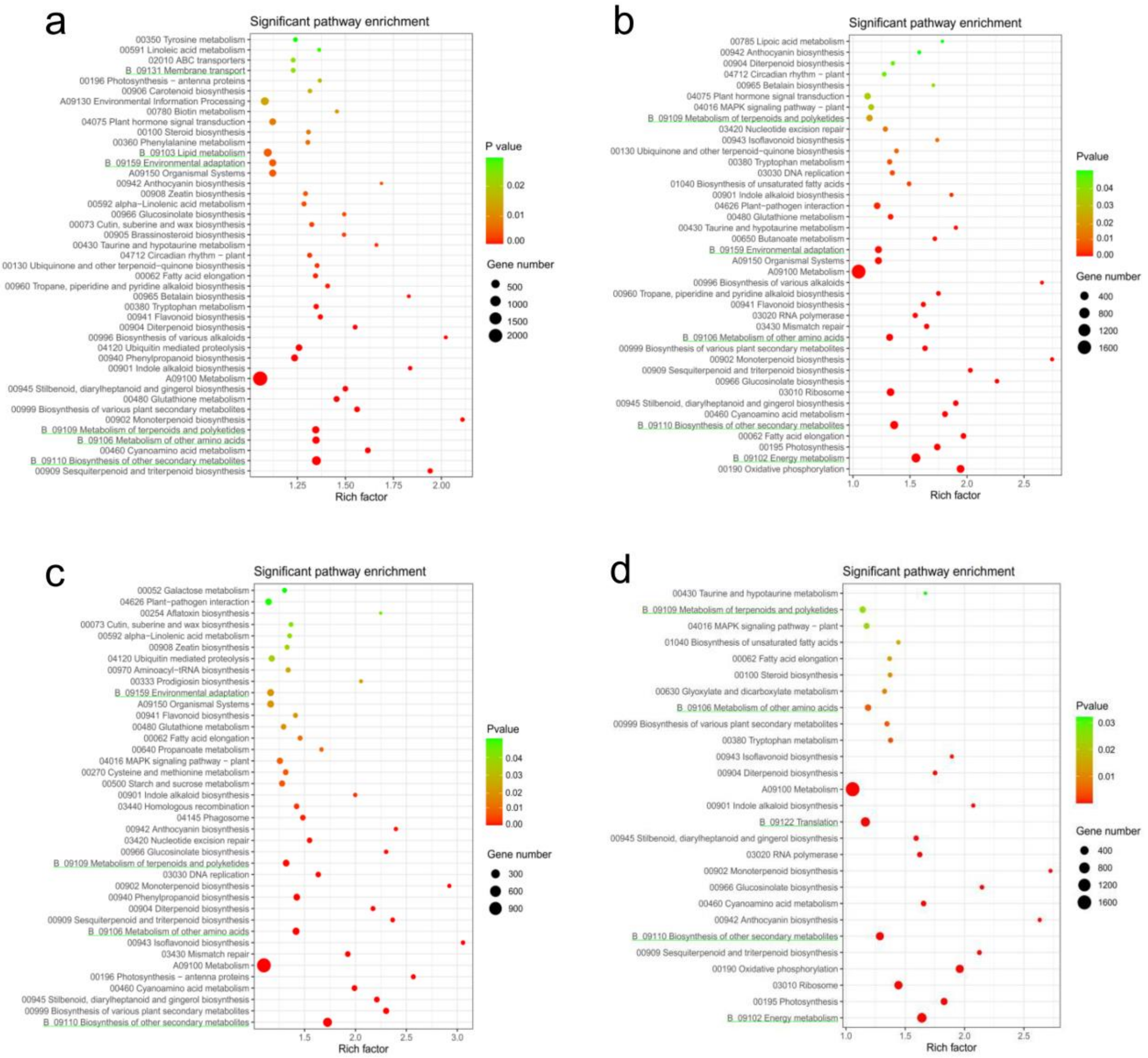

Supplementary Figure S3. KEGG enrichment of expanded genes in four *Avena* species. (**a**) *A. longiglumis* genome. (**b**) *A. atlantica* genome. (**c**) *A. strigosa* genome. (**d**) *A. eriantha* genome.

Supplementary Table S1. Summary of sequencing libraries of *Avena longiglumis* included in the study.

Species	Library	Type	Sample	Configuration	Insertion (bp)	Platform	Read count (Millions)	Total reads bases (bp)	Accession no. in NCBI
A. longiglumis	NDES00415_L4	gDNA	Leaf	2 × 150bp, paired	350	Illumina HiSeq 2500	895.0	268,598,824,800	SRR19279518
Subtotal							895.0	268,598,824,800	
A. longiglumis	20190212-NPL0561-P1-E1-H1	gDNA	Leaf	20–30 kbp	> 23,000	Nanopore PromethION	3.2	48,220,684,593	SRR19279527
A. longiglumis	20190215-NPL0561-P1-E1-H1	gDNA	Leaf	20–30 kbp	> 23,000	Nanopore PromethION	2.7	51,028,783,483	SRR19279522
A. longiglumis	20190216-NPL0561-P1-A3-D3	gDNA	Leaf	20–30 kbp	> 23,000	Nanopore PromethION	1.2	21,004,088,941	SRR19279520
A. longiglumis	20190215-NPL0561-P1-A1-D1	gDNA	Leaf	20–30 kbp	> 23,000	Nanopore PromethION	4.6	84,191,503,177	SRR19279531
A. longiglumis	20190211-NPL0561-P1-E3-H3	gDNA	Leaf	20–30 kbp	> 23,000	Nanopore PromethION	3.6	49,715,012,344	SRR19279525
A. longiglumis	20190212-NPL0561-P2-A7-D7	gDNA	Leaf	20–30 kbp	> 23,000	Nanopore PromethION	3.7	56,313,401,650	SRR19279524
A. longiglumis	20190215-NPL0561-P1-A7-D7	gDNA	Leaf	20–30 kbp	> 23,000	Nanopore PromethION	1.4	24,947,902,936	SRR19279523
A. longiglumis	20190328-NPL0561-P2-E7-H7	gDNA	Leaf	20–30 kbp	> 23,000	Nanopore PromethION	2.1	38,735,683,870	SRR19279528
A. longiglumis	20190329-NPL0561-P1-E1-H1	gDNA	Leaf	20–30 kbp	> 23,000	Nanopore PromethION	2.4	45,977,659,069	SRR19279519
A. longiglumis	20190302-NPL0613-P1-A5-D5	gDNA	Leaf	20–30 kbp	> 23,000	Nanopore PromethION	2.5	38,218,681,407	SRR19279529
A. longiglumis	20190124-NPL0613-P2-A1-D1-ul	gDNA	Leaf	20–30 kbp	> 23,000	Nanopore PromethION	2.1	22,158,343,522	SRR19279526
A. longiglumis	20190221-NPL0613-P2-E1-H1	gDNA	Leaf	20–30 kbp	> 23,000	Nanopore PromethION	1.7	30,848,177,162	SRR19279530
Subtotal							31.2	511,359,922,154	

A. longiglumis	447_18L3A026	gDNA, HiC	Leaf	2 × 150 bp, paired	450	Illumina Novaseq 6000	87.3	26,195,252,100	SRR19279533
A. longiglumis	447_19L3A027	gDNA, HiC	Leaf	2 × 150 bp, paired	450	Illumina Novaseq 6000	75.4	22,623,619,200	SRR19279514
A. longiglumis	B447_5L4A005	gDNA, HiC	Leaf	2 × 150 bp, paired	450	Illumina Novaseq 6000	77.5	23,238,770,100	SRR19279517
A. longiglumis	B447_6L4A006	gDNA, HiC	Leaf	2 × 150 bp, paired	450	Illumina Novaseq 6000	105.9	31,771,530,300	SRR19279516
A. longiglumis	B447_16L4A024	gDNA, HiC	Leaf	2 × 150 bp, paired	450	Illumina Novaseq 6000	77.0	23,104,664,100	SRR19279521
A. longiglumis	B447_18L3A026	gDNA, HiC	Leaf	2 × 150 bp, paired	450	Illumina Novaseq 6000	227.0	68,092,406,400	SRR19279515
A. longiglumis	B447_19L3A027	gDNA, HiC	Leaf	2 × 150 bp, paired	450	Illumina Novaseq 6000	204.3	61,297,082,100	SRR19279532
A. longiglumis	B447_5L3A005	gDNA, HiC	Leaf	2 × 150 bp, paired	450	Illumina Novaseq 6000	207.6	62,277,830,400	SRR19279512
A. longiglumis	B447_6L3A006	gDNA, HiC	Leaf	2 × 150 bp, paired	450	Illumina Novaseq 6000	210.5	63,160,123,500	SRR19279511
A. longiglumis	B447_16L3A024	gDNA, HiC	Leaf	2 × 150 bp, paired	450	Illumina Novaseq 6000	180.0	53,874,397,500	SRR19279513
Subtotal							1452.5	435,635,675,700	
A. longiglumis	ALO_L1	RNA-seq	Leaf	2 × 150 bp, paired	450	Illumina Novaseq 6000	53.5	16,035,567,600	SRR24234795
	ALO_L2	RNA-seq	Leaf	2 × 150 bp, paired	450	Illumina Novaseq 6000	58.0	17,414,147,100	SRR24234796
	ALO_L3	RNA-seq	Leaf	2 × 150 bp, paired	450	Illumina Novaseq 6000	64.2	19,270,228,500	SRR24234797
A. longiglumis	ALO_R1	RNA-seq	Root	2 × 150 bp, paired	450	Illumina Novaseq 6000	60.3	18,089,824,800	SRR24234802
	ALO_R2	RNA-seq	Root	2 × 150 bp, paired	450	Illumina Novaseq 6000	45.4	13,622,818,200	SRR24234803
	ALO_R3	RNA-seq	Root	2 × 150 bp, paired	450	Illumina Novaseq 6000	48.5	14,552,806,200	SRR24234804

Subtotal	329.9	98,985,392,400

Supplementary Table S2. Evaluation of gene space completeness by BUSCO analysis in the *A. longiglumis* genome assembly.

Features	Based on whole genome sequences
Complete BUSCOs (C)	1,597 (99.00%)
Complete and single-copy BUSCOs (S)	1,313 (81.40%)
Complete and duplicated BUSCOs (D)	284 (17.60%)
Fragmented BUSCOs (F)	3 (0.20%)
Missing BUSCOs (M)	14 (0.80%)
Total BUSCO groups searched	1,614

Supplementary Table S3. Statistics of gene function annotation of the *A. longiglumis* genome.

Databases (Full name)	Number	Percentage
NCBI NR (Non-redundant protein)	39,390	96.44%
EggNOG (Evolutionary genealogy of genes: non-supervised orthologous groups)	38,078	93.23%
Pfam (Pfam protein families)	35,573	87.09%
COG (Clusters of orthologous groups)	35,460	86.82%
SwissProt (Swiss Institute of Bioinformatics and Protein Information Resource)	32,099	78.59%
GO (Gene ontology)	21,162	51.81%
KOG (EuKaryotic orthologous groups)	20,756	50.82%
KEGG (Kyoto encyclopedia of genes and genomes)	17,285	42.32%
PlantTFDB (Plant transcription factor)	2,403	5.88%
CAZy (Carbohydrate-Active enZYmes)	931	2.28%
Number of annotated genes	39,558	96.85%
Number of predicted genes	40,845	100%
